# Alteration in Lysophospholipids and Converting Enzymes in Glaucomatous Optic Nerves

**DOI:** 10.1167/iovs.61.6.60

**Published:** 2020-06-30

**Authors:** Sasha M. Milbeck, Sanjoy K. Bhattacharya

**Affiliations:** Department of Ophthalmology, Miami Integrative Metabolomics Research Center, Bascom Palmer Eye Institute, University of Miami, Miami, Florida, United States

**Keywords:** glaucoma, lipidomics, phospholipid metabolism, lysophospholipids, POAG

## Abstract

**Purpose:**

To determine whether lysophospholipid (LPL) profiles and corresponding conversion enzymes in the LPL pathways are altered in the optic nerve (ON) between human control and glaucoma samples.

**Methods:**

Lipids extracted from control (*n* = 11) and glaucomatous (*n* = 12) ON samples using the Bligh and Dyer method were subjected to high-resolution mass spectrometry on a Q-exactive mass spectrometer coupled with a high-performance liquid chromatography (Accela 600) system. Analysis was performed for LPLs (lysophosphatidylcholines, lysophosphatidylserines, lysophosphatidylethanolamines, lysophosphatidylinositols, and lysosphingomyelines) using LipidSearch v.4.1, MZmine v.2.0, and MetaboAnalyst v.4.0. LPL synthesis and degradation pathway maps, utilizing UniProt and BRENDA database entries as needed, were created using Kyoto Encyclopedia of Genes and Genomes (KEGG)–based tools. The mRNA expression level in normal and glaucomatous human ON were analyzed using Gene Expression Omnibus (GEO) entry GSE45570. Protein amounts were determined using PHAST gel and dot blot and were used for normalization of protein amounts across samples. Western blot, ELISA, and protein quantification were performed using established protocols.

**Results:**

Principal component analysis of ON LPL profile placed control and glaucomatous ONs in two distinct separate groups. Mass spectrometric analysis of ON revealed decrease in lysophosphatidic acid, lysophosphatidylethanolamine, lysophosphatidylcholine, and significant increase in diacylglycerol in glaucomatous ON. Statistical analysis of LPL conversion enzymes revealed significant overexpression of phosphatidate phosphatase LPIN2, phospholipid phosphatase 3, phosphatidylcholine-sterol acyltransferase, and calcium-dependent phospholipase 2, and significant downregulation of glycerol-3-phosphate acyltransferase 4 at mRNA level in glaucomatous ON. Western blot and ELISA confirmed proteomic differences between normal and diseased ON.

**Conclusions:**

Our analysis revealed alterations in specific LPL levels and corresponding select enzyme-level changes in glaucomatous ON.

Glaucoma refers to a group of optic neuropathies, which is the second leading cause of blindness worldwide. Glaucoma is characterized by progressive degeneration of retinal ganglion cells (RGCs) in the optic nerve (ON).^1^ RGCs are a group of central nervous system (CNS) neurons that send visual signals from the retina along their axons to the brain. RGC axons pass through the optic nerve head (ONH), an area that is sensitive to structural changes.^2^ In clinical noninvasive studies, optic disc changes and thinning of the nerve fiber layer (NFL) are observed with glaucoma progression using fundus photography and optical coherence tomography, respectively.^3^ The ON undergoes excavation in glaucoma that usually occurs with cumulative exposure to elevated intraocular pressure (IOP) for extended periods. In most forms of glaucoma, an increase in IOP leads to degeneration of RGCs.^4^ RGC cell death is the principal reason for visual loss and ON degeneration in glaucoma.

Lipids are crucial for cell membranes and play important roles in maintaining the integrity of RGC membranes, dendrites, and synapses.^5^^,^^6^ Lipids have great diversity, and it is thought that mammalian cells have >100,000 lipids that are classified into 78 different classes and have diverse biological functions.^7^^–^^9^ Our focus centers around characterizing lipids present in the ON. The integrity of lipids in the ON is critical to the upkeep of important cellular interactions,^10^^,^^11^ and even minor changes in lipid composition has been known to affect functionality of CNS and its neurons.^12^ Aberrations in lipid metabolism in glaucomatous ON could lead to decreased ON stability. Previously we have profiled lipids of the ON and, due to lipids’ tremendous diversity, have focused only on glucosylsphingosine lipids and impairment of sphingolipid enzymatic pathways.^13^ We aim here to investigate lysophospholipids (LPLs) present in the ON.

LPLs are membrane-derived signaling lipid molecules that are produced by enzymatic processes activity of phospholipases on metabolites and lipids, such as phospholipids, sphingolipids, and glycerophospholipids.^14^ LPLs signal through specific G protein-coupled receptors, which are expressed in numerous tissues and cell types, promoting a wide variety of cellular process.^15^^,^^16^ LPLs are intermediate lipid products and various kinds of injury, such as physical pressure, blunt trauma, heat, and chemicals, can lead to their formation.^17^

Although biological membranes, in relative terms to other lipids, comprise very low amounts of LPLs, they have significant biological functions and play important roles in numerous physiological processes.^18^ Changes in LPL metabolism have been found to induce inflammatory responses, apoptosis, cell proliferation, disruptions in mitochondrial functions, and other cellular responses.^19^^–^^23^ Studies conducted on changes in LPL levels’ modulations post-CNS injury suggest that altered LPL production and degradation can disrupt the nervous system and result in neurologic disorders.^24^^–^^26^ Previous studies have investigated the role of LPLs in regulating aqueous humor outflow and IOP,^27^ mechanisms that are impaired in glaucoma, but there is still little knowledge about the role of LPLs in the ON. Studies presented here aim to fulfill that gap. By determining whether aberrations in LPL metabolism exist in glaucomatous ON, we aim to continue investigating the role of LPLs in maintaining necessary mechanisms for healthy vision.

## Materials and Methods

### Tissue Procurement

All human subjects were treated in accordance with the Declaration of Helsinki. Tissues used for lipidomics were obtained from cadaveric donor eyes, which were procured from Eversight Vision Eye Bank (Cincinnati, OH, USA) and Lions Eye Bank (Miami, FL, USA). All donors were Caucasians, inclusive of both sexes, with mean age ± standard deviation of 72.3 ± 5.9 years for control donors, and 70.3 ± 10.5 years for glaucoma donors (Supplementary Table S1). The time for donor eyes’ postmortem to enucleation time ranged from 3 to 21 hours (Supplementary Table S1). The postmortem to enucleation time between control and glaucoma donors showed no significant statistical difference (Supplementary Fig. S3). All eyes were kept in a chamber filled with phosphate buffered saline (PBS) at 4°C. Patients were required to have a clear diagnosis of primary open angle glaucoma (POAG) and have detailed ophthalmic and medical histories. Patients were considered viable glaucoma donors if the patients had at least two static perimetry recordings (within 1–2 years) and showed distinct vision loss over time, as described previously.^13^ Many donor eyes also had measurements of NFL thickness, and other clinical parameters that aid in diagnosis of glaucoma. Acceptable control eyes had no medical history of optic neuropathy. Donor eyes were shipped out immediately, and within 36 hours postenucleation, and on acquiring the eyes dissection was performed. The ON was carefully cut out from ONH, and the fatty tissue surrounding the nerve was removed. Samples were washed with sterile 1X PBS prior to any further analysis. ONs were stored at −80°C until lipidomic analysis. There were 12 out of 40 glaucoma eyes, and 11 out of 27 control donor eyes that were deemed acceptable for lipidomic analysis. Additional donor tissues were utilized for protein quantification, Western blot, and ELISA analysis (Supplementary Table S1).

### Lipid and Protein Extraction

The ON tissue was weighed, minced, and homogenized. A modified Bligh and Dyer method was used to extract the lipids, which was described in our prior reports.^28^ Methanol and chloroform were added in a 2:1 ratio to each sample. Samples were vortexed for 2 minutes followed by 2 minutes of sonication in an ultrasonic bath. Liquid chromatography-mass spectrometry (LC-MS) water (3 mL) and chloroform (1.5 mL) were added and the samples were vortexed. All reagents were of LC-MS grade. To obtain phase separation, the samples were centrifuged at 9800xg at 4°C. The organic phase containing the lipids was collected, dried with argon gas, and stored at −20°C until mass spectrometric analysis. Uniformity of extraction was ensured by adding an extraction standard, usually PC13:0/13:0 (Avanti Polar Lipids, Alabaster, AL, USA) as described in our prior reports.^17^^,^^29^^,^^30^ Prior to mass spectrometric analysis, the dry lipid samples were reconstituted in 50 µL of chloroform to methanol (1:1). Separate donor ON tissue was used for Western blot and ELISA protein analyses. The tissue was homogenized, centrifuged at 10,000 rpm for 10 minutes, and the supernatant was collected. A PHAST gel (GE Healthcare BioSciences AB, Uppsala, Sweden) was performed followed by a densitometry analysis using a bovine serum albumin standard to estimate protein concentrations. The protein amounts were normalized among all the samples.

### High-Performance Liquid Chromatography (HPLC)-Mass Spectrometry

The lipid samples were subjected to liquid chromatography tandem mass spectrometry (LC-MS/MS) analysis. The analysis was performed on an Accela 600 HPLC system connected to a Q Exactive mass spectrometer (Thermo Fisher Scientific, Waltham, MA) equipped with a heated electrospray ionization (HESI) source. Reverse phase HPLC was performed on an Acclaim C30 column (particle size 3.0 µm, 150 × 2.1 mm inner diameter; Thermo Fisher Scientific) with an injection volume of 5 µL and a column temperature of 30°C. The gradient ran from 15% to 65% B for 20 minutes at a flow rate of 260 µL/min. Solvent A was composed of methanol and water (60:40) with 0.2% formic acid and 10 mM ammonium acetate, and solvent B was composed of methanol and chloroform (60:40) with 0.2% formic acid and 10 mM ammonium acetate. The HESI source was operated in positive ionization mode with a spray voltage of 4.4 KV, heated capillary of 350°C, and heater temperature of 275°C. The S-lens radio frequency was set to 70. The gas flow rate was set to 45 units and auxiliary gas to 15 units. The mass range was 200 to 3000 m/z and the resolution for full scan was set to 70,000 and 17,500 for data-dependent ms/ms acquisition. The automatic gain control target was 1 × 10^E6^, and the maximum injection time was 100 ms. The isolation window was set to 1.3 m/z, and the dynamic exclusion to 3 seconds. The stepped normalized collision energies were 19 and 30 eV. At the beginning and end of the run, we ran a fixed amount of a standard mix (usually EquiSPLASH LIPIDOMIX; Catalog number 330731, Avanti Polar Lipids) to ensure the proper functioning of the instruments.

### Lipid Identification, Relative Quantification, and Processing

Raw mass spectrometry files were processed for lipid identification using LipidSearch software version 4.1 (Thermo Fisher Scientific, Tokyo, Japan). For identification, the precursor and product ion search tolerance were 5 ppm; product ion intensity; filters: top rank, main isomer peak, FA priority; quantification: m/z tolerance 5 ppm, retention time tolerance 0.5 minutes. Adducts used in positive mode were +H, +NH_4_, +H-H_2_O, +H-2H_2_O, and +2H_2_+. The samples were run in triplicates and all lipid classes were searched for. The samples rejected by the software were not included in the statistical analysis. For alignment, the retention time was 0.1 minutes, and the top ranked filtered and main isomer peak were selected. All peaks with the same annotated lipid species were merged in the result file. The M-score for the alignments was set to 5, C-score to 2.0, and fatty acids chain identification was set to “A” and “B” allowing the positive identification of the chains. The data exported from LipidSearch 4.1 were grouped by lipid class, fatty acid, and lipid species. The three independent runs for each sample were aligned in the LipidSearch software and only samples with consistent peaks were accepted. We performed relative quantification of the lipids using the peak area following the LipidSearch protocol. The triplicate mean peak intensity values for each lipid class were averaged, and the data were further analyzed in GraphPad Prism 8 (GraphPad, San Diego, CA, USA) and Metaboanalyst 4.0 (Xia Lab at MGill University, Montreal, Quebec, Canada).

### Statistical Analyses

Analyses were conducted using MetaboAnalyst 4.0 and GraphPad Prism 8. For principal component analysis (PCA), data were both quantile normalized and log_2_ transformed to remove skewness and nonbiological, procedure-based variation. In GraphPad Prism, bar graphs were created, and the average peak intensity values were shown as mean ± SE. Outliers were removed using the ROUT method^31^ with Q = 1%. Prior to any statistical analysis, the Shapiro-Wilk test was used to establish normalcy (*P* > 0.05), and the Levene test determined equality of variance. For each lipid class, an unpaired *t*-test with Gaussian distribution was implemented to detect statistical differences between control and glaucomatous LPL classes. Further statistical analysis was performed on the four statistically significant lipid classes to determine if there were peak intensity differences between LPL conversion enzymes in control and glaucomatous ON.

### Genomic Data Analyses

An LPL metabolic pathway was constructed based on knowledge derived from the Kyoto Encyclopedia of Genes and Genomes (KEGG),^32^ BRENDA,^33^ and Uniprot^34^ database. The Gene Expression Omnibus (GEO) entry GSE45570^35^ dataset was used to construct this LPL metabolic pathway. The enzymes found to be upregulated/downregulated in the pathway were confirmed by performing Western blot and ELISA analyses on the 12 ON samples (6 control and 6 POAG).

### Western Blot and ELISA Analyses

Western blot and ELISA analyses were used to detect the enzyme levels in POAG ONs compared with non-POAG controls. The enzymes detected were those found to be upregulated/downregulated in the GEO dataset. For Western blot, equal amounts (10 µL) of protein per sample were fractioned on a 4% to 20% sodium dodecyl-polyacrylamide gel electrophoresis (Invitrogen, Carlsbad, CA, USA). Gel electrophoresis was performed at 80 V for 30 minutes, and 100 V for an additional 45 minutes. The gel was transferred onto a polyvinylidene fluoride (PVDF) membrane using precut blotting sandwiches (Immun-Blot PVDF Membrane Sandwiches, catalog no. 1620238; Bio-Rad, Hercules, CA, USA). The PVDF membranes were blocked with 5% nonfat milk powder in a tris-buffer saline solution of pH 8. The primary antibodies against 1-acyl-sn-glycerol-3-phosphate acyltransferase gamma or AGPAT3 (Proteintech; rabbit polyclonal), Autotaxin or ATX/ENPP2 (Abcam, Cambridge, MA, USA; mouse polyclonal), Glycerophosphodiester phosphodiesterase 1 or GDE1 (Proteintech; rabbit polyclonal), Glyceraldehyde-3-phosphate dehydrogenase or GAPDH (Abcam; mouse monoclonal), Glycerol-3-phosphate acyltransferase 4 or GPAT4 (Proteintech; rabbit polyclonal), Phosphatidylcholine sterol acyltransferase or LCAT (Proteintech, Rosemont, IL; rabbit polyclonal), Lysophosphatidylcholine acyltransferase 1 or LPCAT1 (Proteintech; mouse monoclonal), Phosphatidate phosphatase LPIN2 or LPIN2 (Abcam; rabbit polyclonal), Calcium-dependent phospholipase A2 or PLA2G5 (Thermo Fisher Scientific; mouse monoclonal), and Phospholipid phosphatase 3 or PLPP3 (Thermo Fisher Scientific; mouse monoclonal) were used for protein detection. For Western blot and ELISA analysis, Horseradish peroxidase (HRP) secondary antibodies goat anti-rabbit HRP (1:2000; Abcam) and goat anti-mouse (1:2000; Abcam) were used. Western blot was developed using chemiluminescence reagents on a ImageQuant LAS4000 (Cytiva, Marlborough, MA). ELISA was performed in triplicates using 0.5 µL of extracted protein from control and glaucomatous ON in 96-well plates following established protocols. The absorbance was measured at 450 nm on a plate reader. The absorbance of each protein was normalized to GAPDH amounts in control and glaucomatous ON, respectively. An unpaired *t*-test was run to determine differences in absorbance between control and glaucomatous samples.

## Results

**Figure 1.**
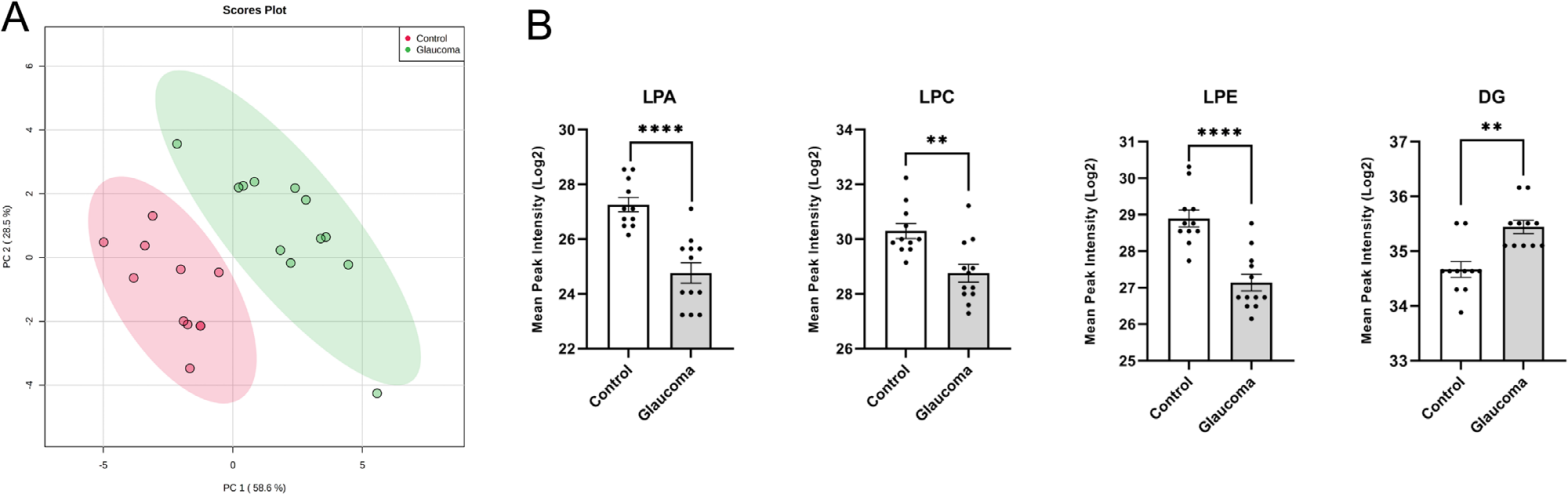
Analysis of principal component and amounts of different LPLs of the ON of cadaveric human control and glaucomatous ON. (**A**) PCA of total LPLs of ON was created using Metaboanalyst 4.0. Mean peak intensity mass spectrometric data were quantile normalized and log2 transformed. The control and glaucoma group symbols are as indicated. (**B**) Mean peak intensity (log2 transformed, quantile normalized) of LPA, LPC, LPE, and DG in glaucomatous versus normal control samples determined from mass spectrometric data as indicated. For each lipid class, we performed an unpaired parametric Student's *t*-test between control and glaucoma (****P < 0.001, **P < 0.02).

The PCA of LPLs of 11 cadaveric control (average age: 72.3 ± 5.9 years; 6 men, 5 women) and 12 glaucomatous (average age: 70.3 ± 10.5 years; 8 men, 4 women) ONs (total of 23 human cadaveric eyes; Supplementary Table S1) presented two distinct clusters (Fig. 1A). The scores for the first two principal components, PC1 and PC2, explained the variance, indicating that glaucomatous and control ONs have significantly different LPL classes. These distinct clusters also showed the similar distinct clusters on partial least square discriminant analysis (Supplementary Fig. S4) and provoked our interest into LPL differences between glaucomatous and healthy control ON.

**Figure 2.**
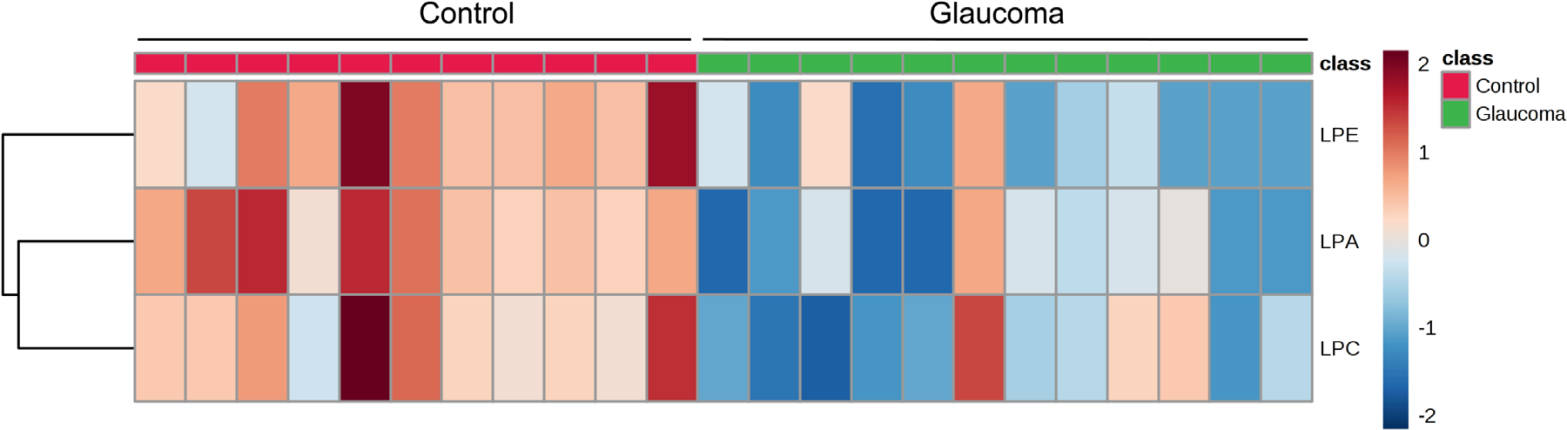
Heatmap of LPE, LPA, and LPC. The heatmap was created using Metaboanlyst 4.0. Mass spectrometric data were quantile normalized and log2 transformed and has been organized by group (control and glaucoma). Analysis parameters included autoscaling based on samples, Ward clustering algorithm, and Euclidean distance measure.

**Figure 3.**
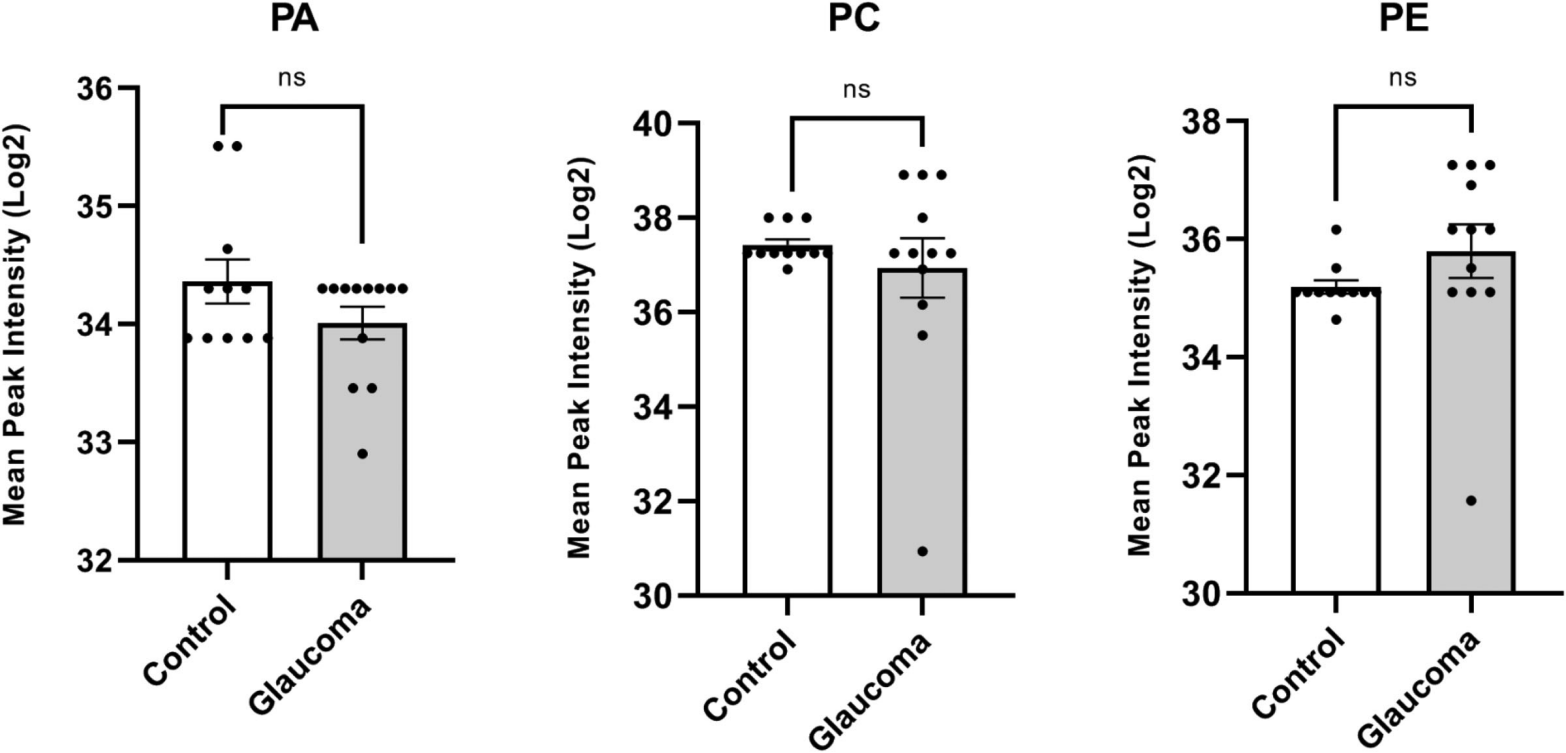
Mean peak intensities of PA, PC, and PE between human control and glaucomatous ON. Quantile normalized and log2 transformed mass spectrometric mean peak intensities were used to estimate relative abundance between control and glaucomatous ON as indicated. An unpaired parametric Student's *t*-test between control and glaucoma (with Gaussian distribution) was performed for each lipid class to determine significance.

We next analyzed the mass spectrometric data for species of LPLs: lysophosphatidic acid (LPA), lysophosphatidylcholine (LPC), lysophosphatidylethanolamine (LPE), lysophosphatidylethanol (LPEt), and lysodimethylphosphatidylethanolamine (LdMePE). The mean peak intensity values of LPA, LPE, and LPC were significantly lower in glaucomatous ON (Fig. 1B). The results of the *t*-test concluded that there were differences between control and glaucomatous ON lipid amounts in all searched LPL classes. A heatmap showed that the differences in control and glaucomatous ON are representative and shared by a majority of samples in each group (Fig. 2). We also determined the relative abundance of precursor species of LPLs, namely phosphatidic acid (PA), phosphatidylcholine (PC), phosphatidylethanolamine (PE), and a related species diacylglycerol (DG) (Fig. 3). Although none of the precursor phospholipids showed statistically significant differences between control and glaucomatous ON (Fig. 3), there was a significant increase in DG in glaucomatous ON (Fig. 1B). Based on these findings, we decided to focus our study on LPA, LPC, and LPE and their interconnected metabolic pathways.

**Figure 4.**
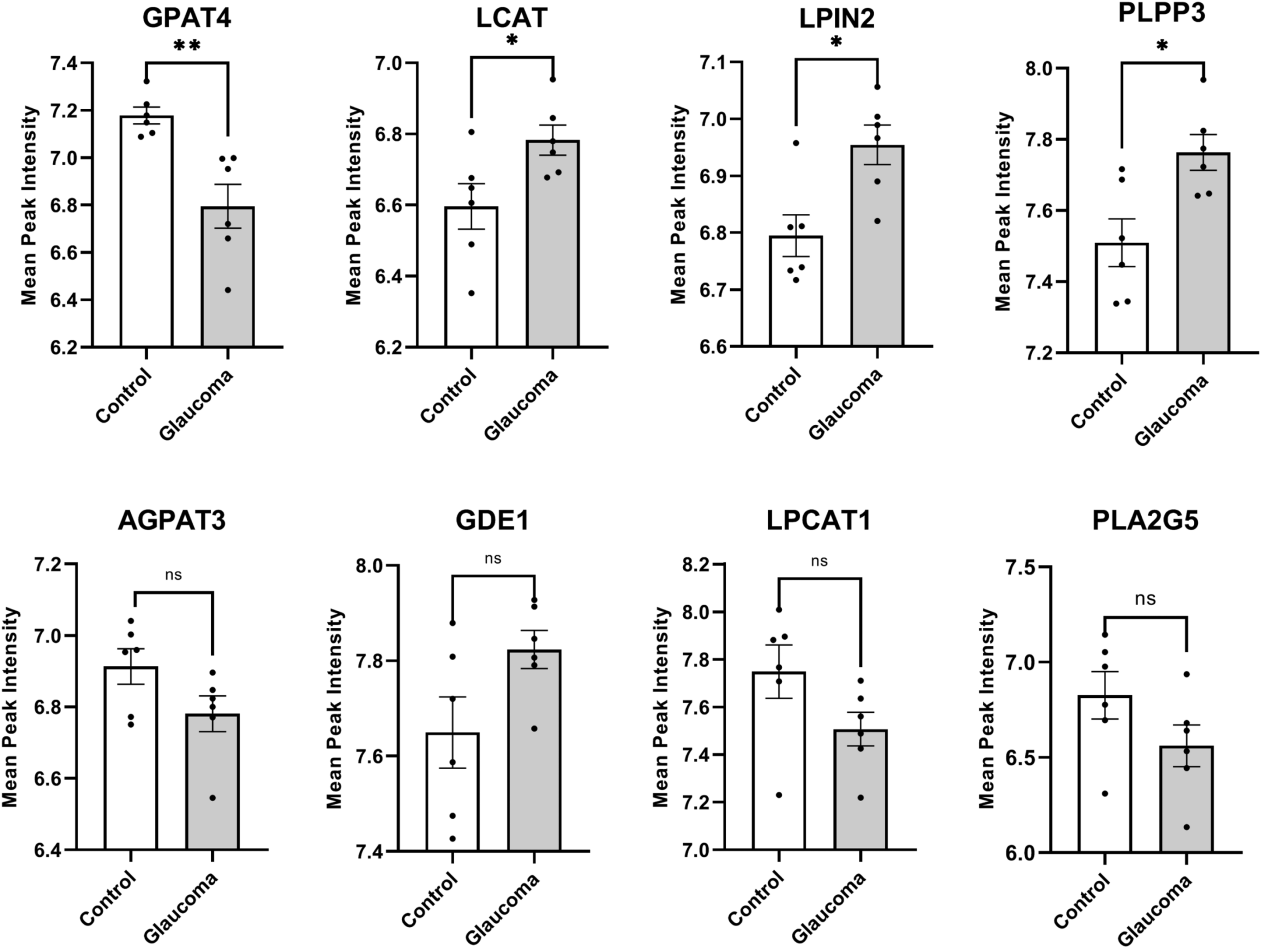
Evaluation of mRNA expression levels of LPL conversion enzymes in glaucomatous and control ON samples. The GEO dataset GSE45570 was used for these analyses. The dataset provided mRNA amounts for each protein in (*n* = 6) control and (*n* = 6) POAG ONHs. The RNA amount for genes of interest was obtained for each sample and averaged across control and glaucomatous samples, respectively. A significant overexpression of LPIN2, PLPP3, and LCAT, and a significant downregulation of GPAT4 protein corresponding mRNA was found in glaucomatous samples. We performed an unpaired parametric Student's *t*-test with Gaussian distribution between control and glaucoma (**P < 0.01; *P < 0.02).

Several enzymes are involved in the synthesis and degradation of LPA, LPC, and LPE. The synthesis, degradation, and interconversion of these lipid species is highly interconnected and rather complicated (Supplementary Fig. S1). To analyze level differences in catalytic enzymes, we used genomic analysis of mRNA to serve as guidance to pursue further actual proteome-level changes in these enzymes. We used genomic data (GSE45570) to determine the relative mRNA amounts of each enzyme in control compared with glaucomatous ON. Our analysis found four enzymes with significant differences: LPIN2, LCAT, PLPP3, and GPAT4 between control and glaucomatous ON (Fig. 4). LCAT, LPIN2, and PLPP3 levels were significantly increased, whereas GPAT4 levels were significantly decreased in glaucomatous ON (Fig. 4). We also evaluated the differences in PLA2G5 and autotaxin (ENPP2 or ATX). Our analysis showed lack of significant changes in PLA2G5 or ATX mRNA levels in glaucomatous ON compared with controls (Fig. 4).

**Figure 5.**
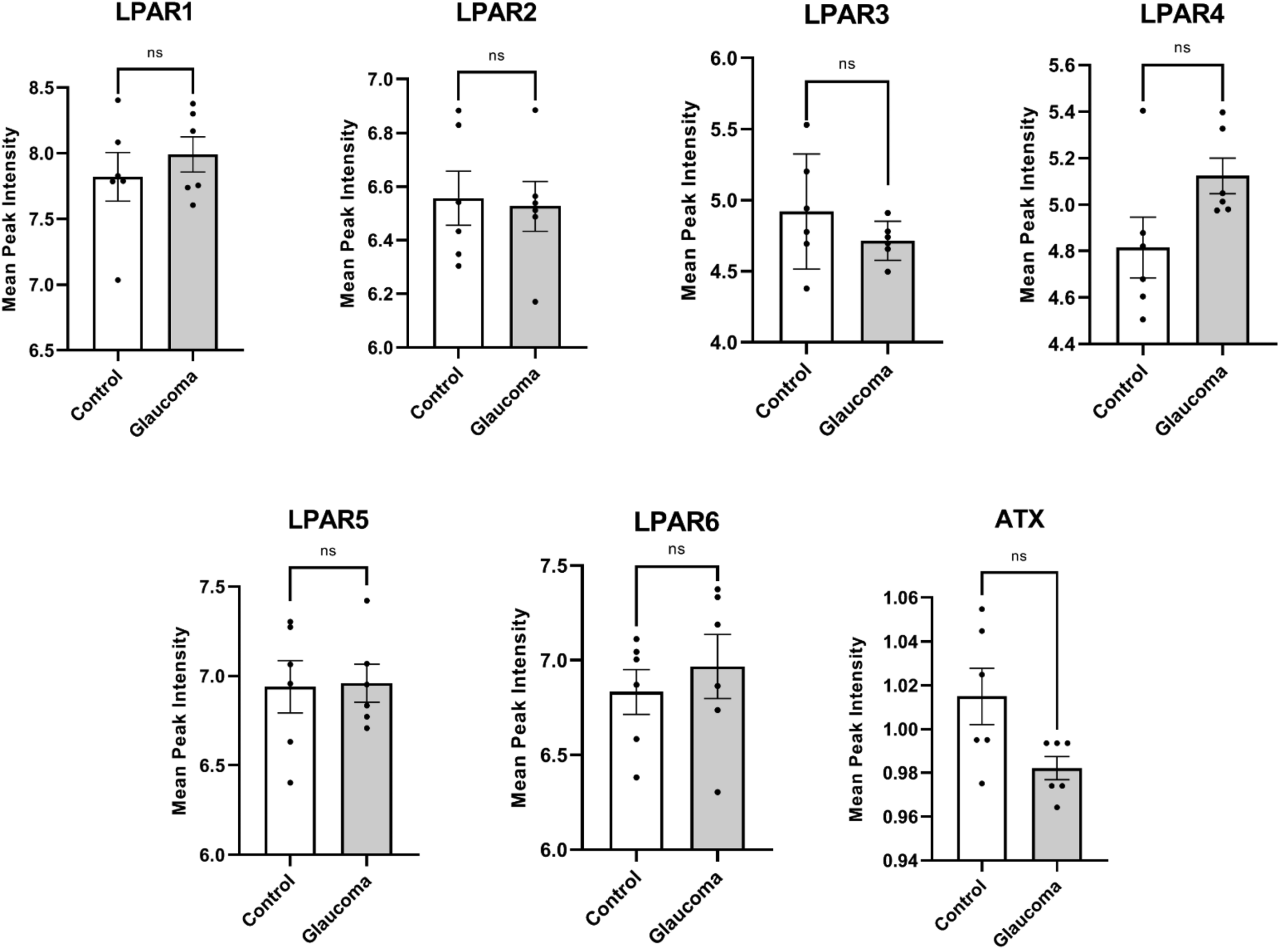
Analysis of genomic data (GSE45570) for all six LPARs and ATX. There was no statistically significant difference in the amount of LPAR 1-6 or ATX mRNA in glaucomatous ON compared with controls. We performed an unpaired parametric Student's *t*-test between control and glaucoma to test for significance.

Because LPA is one of the significantly reduced LPLs in glaucomatous ON, known to have pleotropic effects and analysis of genomic data showed changes in enzymatic levels, we next evaluated lysophosphatidic acid receptors (LPAR) 1-6 mRNA levels as well to better understand if receptors are also altered in concert with enzyme levels. However, our analysis showed no significant difference between any of the LPAR receptors (Fig. 5).

**Figure 6.**
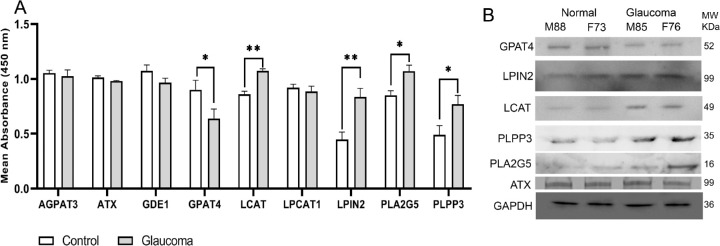
Quantitative estimates of enzymatic proteins in the control and glaucomatous ON. (**A**) ELISA analysis. ON proteins (0.5 µg) with antibodies specific to enzymatic proteins as indicated. Mean absorbance at 450 nm was determined after incubating with secondary antibodies following established protocols. We performed an unpaired parametric Student's *t*-test between control and glaucoma (**P < 0.02, *P < 0.05). (**B**) Representative Western blot analysis. ON proteins (5 µg) were transferred on to PVDF membrane and probed with antibodies specific to enzymatic proteins as indicated.

We performed quantitative ELISA (Fig. 6A) and Western blot analysis (Fig. 6B) to determine whether protein levels are in conformity with mRNA levels (Fig.4). The control and glaucomatous ON protein extracts were used to determine the protein levels of ATX or ENPP2, LPIN2, LCAT, PLA2G5, PLPP3, and GPAT4 (Figs. 6A, 6B). In GAPDH normalized ELISA (Fig. 6A) and Western blots (Fig. 6B), there were elevated levels of LCAT, LPIN2, PLA2G5, and PLPP3 in glaucomatous ON, and decreased levels of GPAT4. The Western blot and ELISA analyses were consistent with the genomic data. The ATX or ENPP2 showed no significant difference between control and glaucomatous ON, respectively (Figs. 6A, 6B). We also performed ELISA analysis for AGPAT3, GDE1, and LPCAT1. There were differences in mRNA levels of these enzymes, although they were not statistically significant. However, due to differences in mRNA levels we decided to perform additional experiments. ELISA analysis confirmed that the protein levels of these enzymes in glaucoma are comparable to controls.

**Figure 7.**
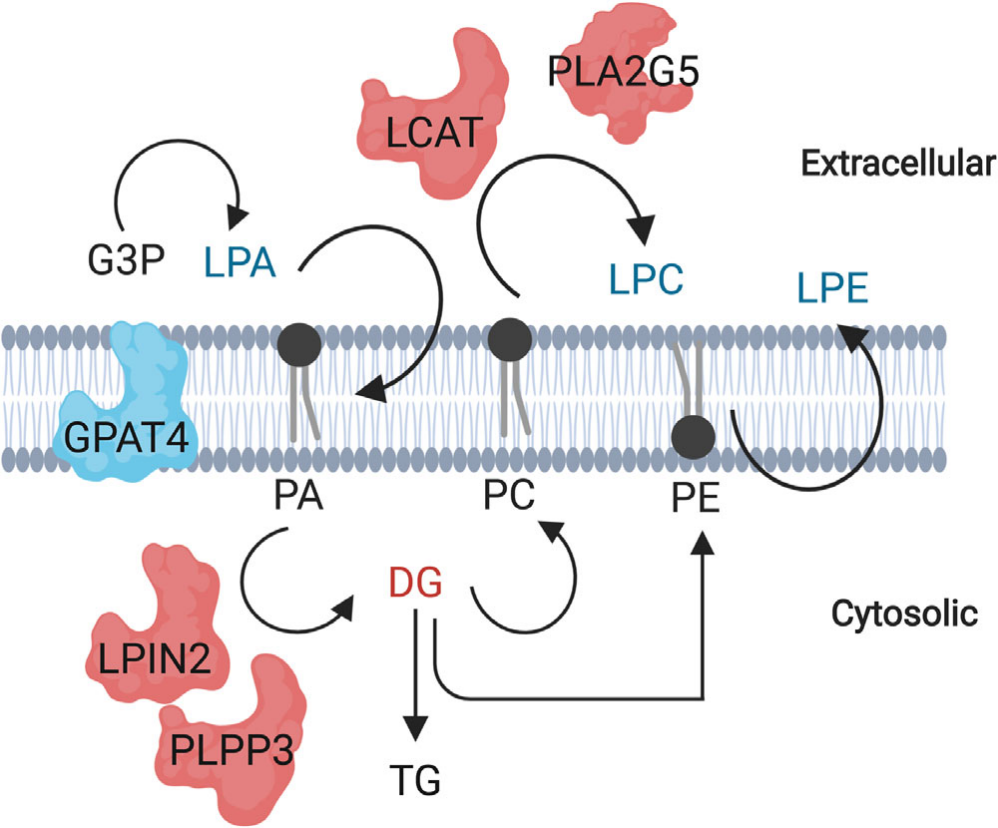
A schematic diagram depicting our findings of the aberrations present in the metabolism of LPA, LPE, and LPC. A depiction of how the pathways of the respective LPLs are related, and the enzymes of interest that help catalyze each metabolic reaction. The enzymes depicted in *red* are upregulated in glaucoma, whereas the enzymes depicted in *blue* are downregulated. PLA2G5 and LCAT are extracellular, whereas GPAT4, LPIN2, and PLPP3 are cytosolic.

We have presented a schematic depiction to summarize our findings (Fig. 7). The nonbilayer lipids, LPA, LPC and LPE, shown in blue are downregulated in glaucoma; DG, a precursor to certain phospholipids, is shown in red and is upregulated in glaucoma, whereas the lipids whose levels were unchanged are in black, including bilayer lipids PC and PE. The enzymes depicted in red are upregulated in glaucoma, for example LPIN2 and PLPP3, whereas the GPAT4 depicted in blue are downregulated. PLA2G5 and LCAT are extracellular, whereas GPAT4, LPIN2, and PLPP3 are found in cytosolic side whether bound to specific or multiple organelles.

## Discussion

LPLs are nonbilayer lipids.^36^ They may be generated owing to processes consistent with biosynthesis,^37^ but they may be generated in processes that are consistent with degradation or lysis of lipids,^17^ including bilayer lipids. LPLs generated in several tissues are commensurate with increased pressure on membranes and/or owing to inflammation.^37^^,^^38^ Glaucoma is associated with elevated IOP,^39^^,^^40^ and inflammation^41^ has also been linked to pathophysiology of glaucoma. Signaling mediated by LPLs has been thought to play a critical role in the physiology of IOP homeostasis.^27^ LPL signaling mediated by LPA also plays a critical signaling role in the nervous system. The six LPA and five sphingosine 1-phosphate and their lipid legends are involved in regulation of various functions in the CNS.^42^ Lipid metabolism assumes a central position in many neuronal diseases that are progressive in nature. Lipidomics studies have proven to provide significant insight for neurologic disorders.^43^ To further study the role of lipids in CNS diseases, we have performed high-resolution mass spectrometry on human ON extracted lipids.^13^ We analyzed glucosylsphingosine lipids in the ON in our previous studies, and due to lack of any comprehensive studies on ON LPLs, we chose to focus here on LPLs for their importance in neuronal signaling, function, and pathophysiology.^37^^,^^38^^,^^42^ Analysis of mass spectrometry lipidomic data revealed a decrease in levels of LPA, LPC, and LPE in the human glaucomatous ON compared with control (Fig. 1B). The decrease is consistent with individual ONs in glaucoma (Fig. 2). We also found statistically significant increase in levels of DG in human glaucomatous ON (Fig. 1B), which is explained by the overexpression of LPIN2 and PLPP3 (Fig. 4), both enzymes that catalyze the formation of DG from PA. Our results demonstrated increased levels of LPEt and LdMePe lysolipids, but their levels were not consistently increased in all individual ONs (Supplementary Fig. S2). We did not find statistically significant differences in the levels of PA, PC, and PE in the glaucomatous ON compared with controls (Fig. 3). Our data indicated decreased levels of PE but not that of PC in the glaucomatous ON. The PC is a major lipid constituent of the lipid bilayer, thus even if a small fraction of it is converted into LPC, we expect little appreciable changes in the concentration or estimated total amount of PC in the ON (Fig. 3). Thus despite a decrease in LPC content (Fig. 1B), alteration in PC is insignificant (Fig. 3). In contrast, a decrease in LPE (Fig. 1B) is perhaps reflected in increased PE level in the glaucomatous ON (Fig. 3).

Previous studies have investigated the changes in LPLs, and have noted a significant increase in LPA in glaucomatous trabecular meshwork tissue/cells and aqueous humor due to an impairment in the ATX-LPA axis.^44^^,^^45^ Models of neuronal diseases have associated an increase of LPC, LPA, and ATX with neuropathic pain and neuronal death.^46^^,^^47^ Despite a decrease in level of LPA (Fig. 1B), we did not observe any appreciable increase in the level of ATX (ATX or ENPP2) levels either using ELISA, Western blot (Figs. 6A, 6B), or using dot blot analysis (Supplementary Fig. S5). An increase in LPA has also been seen to induce RGC death and growth cone collapse in chick embryos.^48^ These findings suggest an aberration in the LPA-ATX pathway to be a cause for the prognosis of many neuronal disorders. Our mass spectrometric analysis revealed a significant decrease in LPA and other LPL classes in glaucomatous ON (Fig. 1B). The drastic differences in LPL amounts between tissues (trabecular meshwork in the anterior eye chamber vs. ON) important in the pathophysiology of glaucoma sparked our interest in studying LPL metabolism in glaucomatous ON. Our genomic (Fig. 5) and proteomic (Fig. 6) analyses have shown that the amounts of ATX, an enzyme responsible for the conversion of LPC to LPA, remains unaltered in glaucomatous ON. Given the importance of LPA signaling in the nervous system and because of the decreased LPA in human glaucomatous ON (Fig. 1), we looked into the mRNA levels of all six LPARs, only to find that their levels are relatively equal between normal and glaucomatous ON (Fig. 5). Our findings are inconsistent with aberration in the LPA-ATX pathway in the glaucomatous ON, unlike that observed in the anterior eye chamber/trabecular meshwork.^44^^,^^45^^,^^49^^–^^51^

Aside from the ATX-LPA pathway, there are numerous metabolic mechanisms that are responsible for the synthesis and breakdown of LPLs. We evaluated several pathways that interconnect the metabolism of LPA, LPE, and LPC (Supplementary Fig. S1) and looked into numerous conversion enzymes that could be responsible for the decrease in the LPLs. This was one of the reasons that we recognized the limitations of availability of authenticated reagents for probing these interconnected pathways at protein levels and considered that genomic level evaluation may help guide toward a narrowed list of enzymatic/protein entities for follow-up. Genomic analysis of human ON revealed impairment in four enzymes of interest: GPAT4, LCAT, LPIN2, and PLPP3 (Fig. 4). We next performed ELISA (Fig. 6A) and Western blot (Fig. 6B) analysis, based on a guided genomic approach. Our experiments supported the genomic analysis and confirmed that the four enzyme (GPAT4, LCAT, LPIN2, PLPP3) protein levels are being aberrant in POAG (Figs. 4, 6), along with additional aberrant level of PLA2G5. The dot blot analysis was found consistent with Western blot and ELISA (Supplementary Fig. S5).

GPAT4 and LIPIN2 are part of the glycerol-3-phosphate pathway, in which the de novo synthesis of glycerol 3 phosphate (G3P) occurs through a stepwise addition of activated fatty acyl groups, each step being catalyzed by distinct enzymes.^52^ The G3P pathway relies on GPATs, AGPATs, LIPINs, and DGATs. We investigated all enzymes in these families (at genomic/mRNA level) and found GPAT4 and LIPIN2 to be the only enzymes with statistically significant differences between control and glaucoma.

GPAT4 was the only enzyme found to be significantly decreased in POAG ON (Figs. 4, 6). GPAT4 is part of the G3P acyltransferase family and converts glycerophosphoric acid into LPA. A decrease in GPAT4 supports the hypothesis that less LPA is being formed in glaucomatous ON. GPAT is the first rate-limiting enzyme in the glycerolipid synthesis pathway, and has the lowest specific activity.^53^ A decrease in GPAT4 has been shown to decrease LPA and PA production: mechanisms necessary for production of triglyceride (TG) and other LPLs. However, although studies have shown that an overexpression of GPAT4 can lead to accumulation of LPA, PA, and certain species of PC, TG levels did not rise.^54^^,^^55^ This suggests that LPA and PA produced from the GPAT4 pathway may be different from the LPA and PA used for TG synthesis.^56^ Our mass spectrometric data suggests that there are insignificant differences in TG levels between control and glaucomatous ON.

PLPP3, a member of the LPP (lysolipid phosphatase) family, has been suggested to limit LPA signaling, degrades extracellular LPA, and inhibits the function of G-protein-coupled receptors for LPA.^57^^,^^58^ Based on our results, there are similar mRNA levels of LPARs in glaucomatous ON (Fig. 5). It is possible that PLPP3 function in the glaucomatous ON occur through mechanisms independent of known LPA receptors.^59^ Compared with other LPP enzymes, PLPP3 has the highest selectivity for LPA, and promotes hydrolysis of extracellular LPA.^60^^,^^61^ An increase of PLPP3 in glaucomatous ON explains the decrease of LPA (Fig. 1B).

PLPP3, as well as LPIN2, were found to be increased in glaucomatous ON (Fig. 6). These enzymes reside on the cytosolic rather than extracellular side of the cells (Fig. 7). LPIN2 is a member of the phosphatidate phosphatase family, which catalyzes the dephosphorylation process of PA to form DG for TG and phospholipid (PC, PE, and PS) synthesis. A recent study^62^ showed an increase in LPIN1, a member of the LPIN family, to suppress axonal regeneration. Knocking out LPIN1 led to a significant decrease in TG, and a significant increase in PC and PE. On LPIN1 deletion, storage lipids were lower, and membrane lipids were higher in neurons, indicating that a deletion of LPIN1 can contribute to membrane integrity and axonal regeneration. LPIN1 is a close family member of LPIN2, an enzyme we have found to be significantly increased in glaucomatous ON (Figs. 4, 6). A similar mechanism could be occurring in glaucomatous ON in which LPIN2 amounts increase post-ON injury. In future studies, depletion of LPIN2, as well as PLPP3, LCAT and PLA2G5 (Figs. 4, 6; the other enzymes increased in glaucomatous ON), will expand our understanding into ON damage in POAG and their synergistic potential for regeneration.

There is an active interest in potential targets for stimulating RGC growth and axon regeneration^63^ that can restore vision in POAG and others suffering from glaucomatous or traumatic injuries to ON. Because of the growth-inhibitory environment of the CNS, and RGCs intrinsic inability to recover from injury, finding a successful mechanism for ON regeneration has been incredibly difficult.^64^

However, only a few studies have thus far considered lipids or lipid metabolism pathways as potential agents for regeneration. Targeting glycerolipid metabolism has been one of the successful avenues for long-distance regeneration.^62^^,^^65^ We expect to learn lessons from studying degenerating ON LPLs that can potentially be utilized for regeneration. Our studies here show an elevation of LPIN2, and recently a reduction of LPIN1, a related protein with similar enzymatic activity, has been shown to promote long-distance ON neuron regeneration.^62^

In summary, our results show interconnected LPL metabolism in the ON and the aberrations present in glaucomatous ON (Fig. 7). Our findings presented here are a first step in studying the effects of LPL decrease on the degeneration of the ON and how a reversal can potentially be used to promote ON neuron regeneration. Future investigations will focus on localizing the aberrations to specific cells in the ON and identifying a mechanism to restore LPL loss in glaucomatous ON.

## Conclusions

Relative abundance of LPA, LPE, and LPC was significantly decreased, and relative abundance of DG was significantly increased in glaucomatous ON, compared with healthy controls. Genomic and protein analysis identified that LCAT, LPIN2, PLA2G5, and PLPP3 were significantly overexpressed in glaucomatous ON, whereas GPAT4 was significantly downregulated in glaucomatous ON. Future studies will identify additional aberrations in the lysolipid metabolism pathways in glaucomatous ON.

## Supplementary Material

Supplement 1

Supplement 2
