# Supplementary material for: Alteration in Lysophospholipids and Converting Enzymes in Glaucomatous Optic Nerves
Source: Invest Ophthalmol Vis Sci. 2020 Jun 30;61(6):60. doi: 10.1167/iovs.61.6.60 (PMC7415893; doi:10.1167/iovs.61.6.60)
Supplement: Supplement 1 [file iovs-61-6-60_s001.pdf]

Supplemental Table 1: Optic Nerve donors§

Donors used for Mass spectrometry

Sample Number	Age	Gender	Time interval*	Cause of Death	MD	PSD (dB)	IOP (mmHg)	Max known IOP (mm Hg)
			Control					
1N	78	M	8	Cardiac Arrest				
2N	78	M	9	Cardiac Arrest				
3N	69	F	11	Cardiac Arrest				
4N	69	M	12	Not known				
5N	65	M	14	COPD				
6N	64	M	10	COPD				
7N	77	F	12	Cardiac Arrest				
8N	78	F	9	Cardiac Arrest				
9N	80	F	10	Trauma				
10N	68	M	6	COPD				
11N	70	F	9	Cardiac arrest				
			Glaucoma					
1G	75	M	3	Cardiac arrest	-2.56 dB	2.54	14	22
2G	70	M	7	Subarachnoid hemorrhage	-4.93 dB	3.23	15	21
3G	68	M	6	Esophageal Cancer	-13.72 dB	9.52	17	27
4G	68	M	8	Lung Cancer	-6.44 dB	5.54	16	23
5G	69	F	19	Myocardial Infarction	-15.59 dB	9.54	19	30
6G	52	M	10	Not known	-3.96 dB	3.72	17	24
7G	82	F	21	COPD	-3.5 dB	2.52	14	22
8G	81	F	9	Ovarian Cancer	-6.9 dB	5.66	15	24
9G	79	M	19	Myocardial Infarction	-4.6 dB	3.96	16	22
10G	65	M	11	Not known	-2.2 dB	2.22	14	22
11G	83	F	10	Not known	-13.90 dB	9.55	17	26
12G	52	M	16	Myocardial Infarction	-6.55 dB	4.58	15	23

Western blot and ELISA analysis

Sample Number	Age	Gender	Time interval*	Cause of Death	MD	PSD (dB)	IOP (mmHg)	Max known IOP (mm Hg)
			Control					
12N	66	M	10	Not known				
13N	71	F	9	Cardiac Arrest				
1N	78	M	8	Cardiac Arrest				
2N	78	M	9	Cardiac Arrest				
3N	69	F	11	Cardiac Arrest				
4N	69	M	12	Not known				
101N	88	M	8	Cardiac Arrest				
102N	73	F	9	Cardiac Arrest				
			Glaucoma					
13G	65	M	8	Myocardial Infarction	-4.55 dB	3.33	16	23
14G	73	F	9	Cancer	-6.66 dB	5.52	16	25
1G	75	M	3	Cardiac arrest	-2.56 dB	2.54	14	22
2G	70	M	7	Subarachnoid hemorrhage	-4.93 dB	3.23	15	21
3G	68	M	6	Esophageal Cancer	-13.72 dB	9.52	17	27
4G	68	M	8	Lung Cancer	-6.44 dB	5.54	16	23
301G	85	M	10	Cardiac Arrest	-4.22	3.22	18	24
302G	76	F	11	Breast cancer	-6.46	4	16	22

*Time interval refers time from death to preservation.§Eyes were preserved in phosphate buffered saline upon enucleation and stored at 2-8ºC during transportation. The glaucoma donors were selected to avoid those who had hyperlimidemia and/or were on statins. Shaded area are donors who are common between mass spectrometry and biochemical experiments.
