# Supplementary material for: Alteration in Lysophospholipids and Converting Enzymes in Glaucomatous Optic Nerves
Source: Invest Ophthalmol Vis Sci. 2020 Jun 30;61(6):60. doi: 10.1167/iovs.61.6.60 (PMC7415893; doi:10.1167/iovs.61.6.60)
Supplement: Supplement 2 [file iovs-61-6-60_s002.pdf]

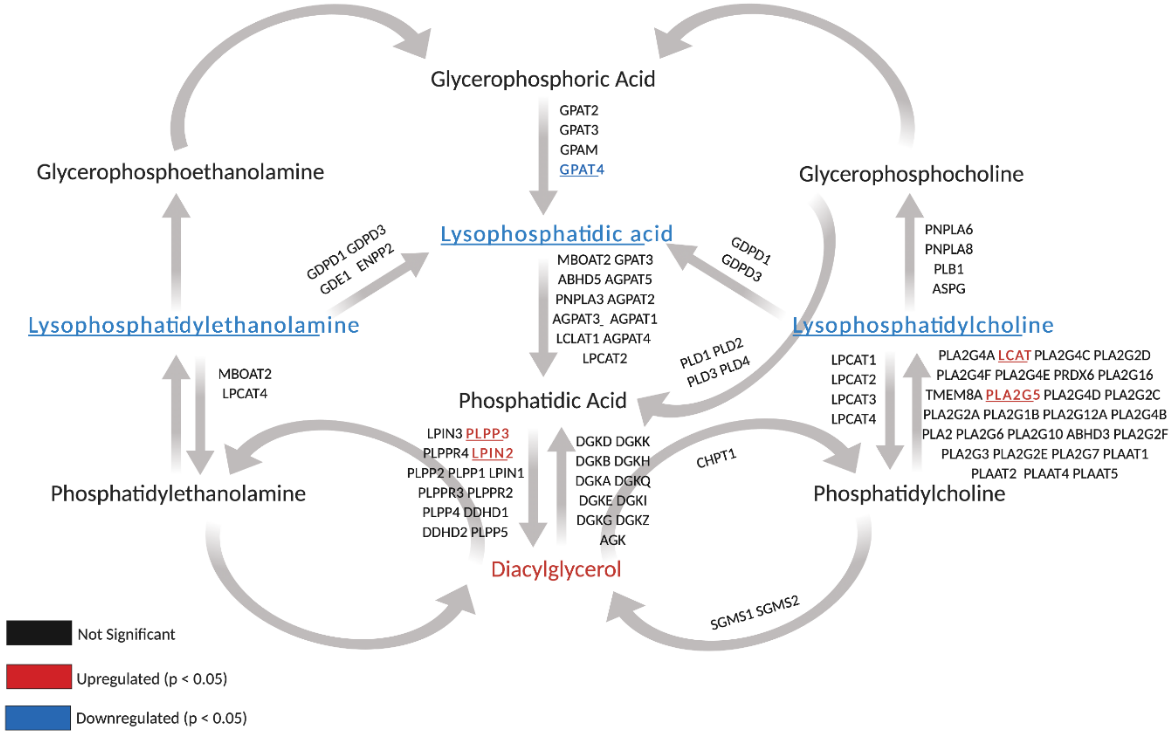

Supplemental Figure 1. Composite lysophospholipid metabolism pathway. A combined diagram containing the findings regarding the pathways of synthesis and degradation of lysophosphatidic Acid (LPA), lysophosphatidylcholine (LPC), and lysophosphatidylethanolamine (LPE). The diagram depicts how the pathways of the respective lysolipids are related, and the enzymes that help catalyze each synthesis and degradation reaction. The enzymes depicted in red are upregulated in patients with Primary Open Angular Glaucoma (POAG), the enzymes depicted in blue are downregulated in patients with POAG, and the enzymes depicted in black were considered to not have a notable difference ($p > 0.05$) between glaucomatous and normal samples. Enzyme names shown are abbreviated from UniProt.

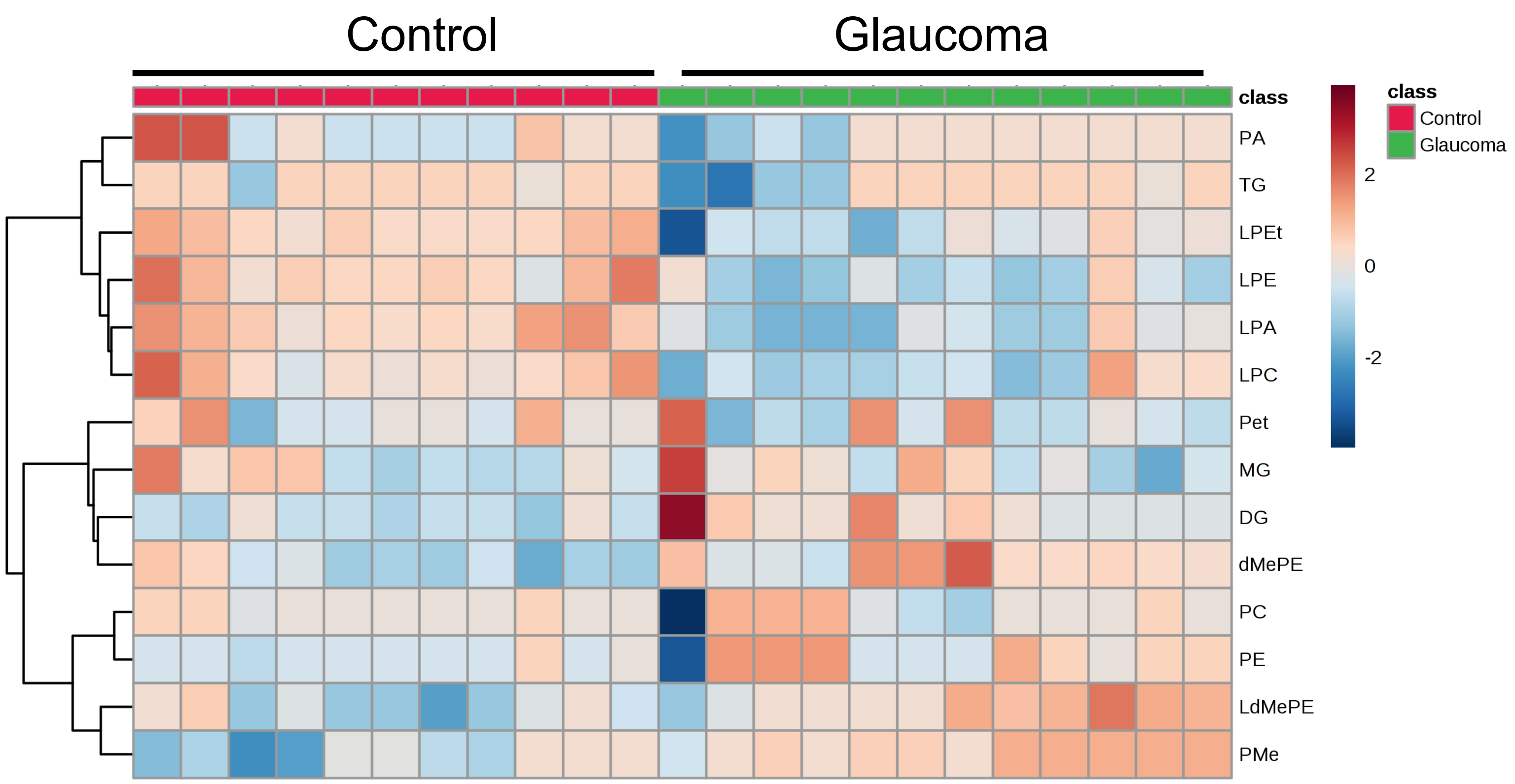

Supplemental Figure 2. Heatmap of lysophospholipids (LPC, LPA, LPE, LPEt, LdMePe), phospholipids (PA, PC, PE, Pet, PMe, dMePe), and glycerides (DG, MG, TG) between cadaveric human control and glaucomatous ON. Data has been organized by group (control versus glaucoma as indicated). Analysis parameters utilized were quantile normalization and log2 transformation of data, Ward clustering algorithm, and Euclidean distance measure.

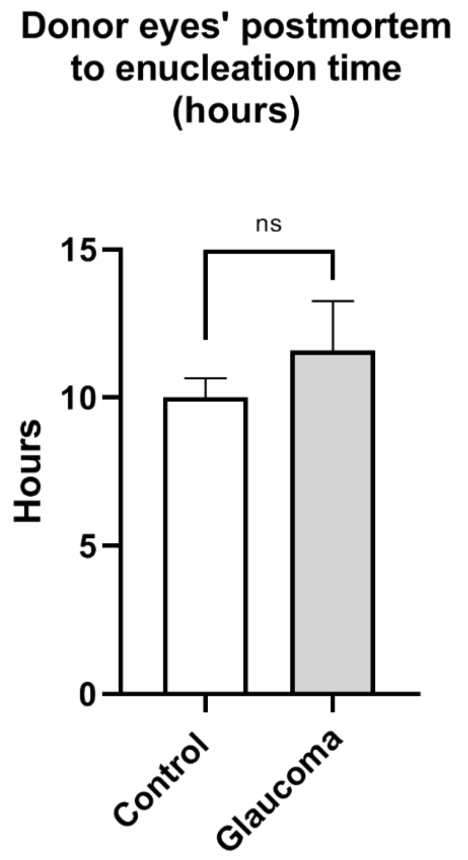

Supplemental Figure 3. Donor eyes' postmortem to enucleation time. There was not a statistical difference in acceptance time between control and glaucomatous donors' optic nerves. The average postmortem to enucleation time for control donors was 10 hours, and 11.6 hours for glaucoma donors.

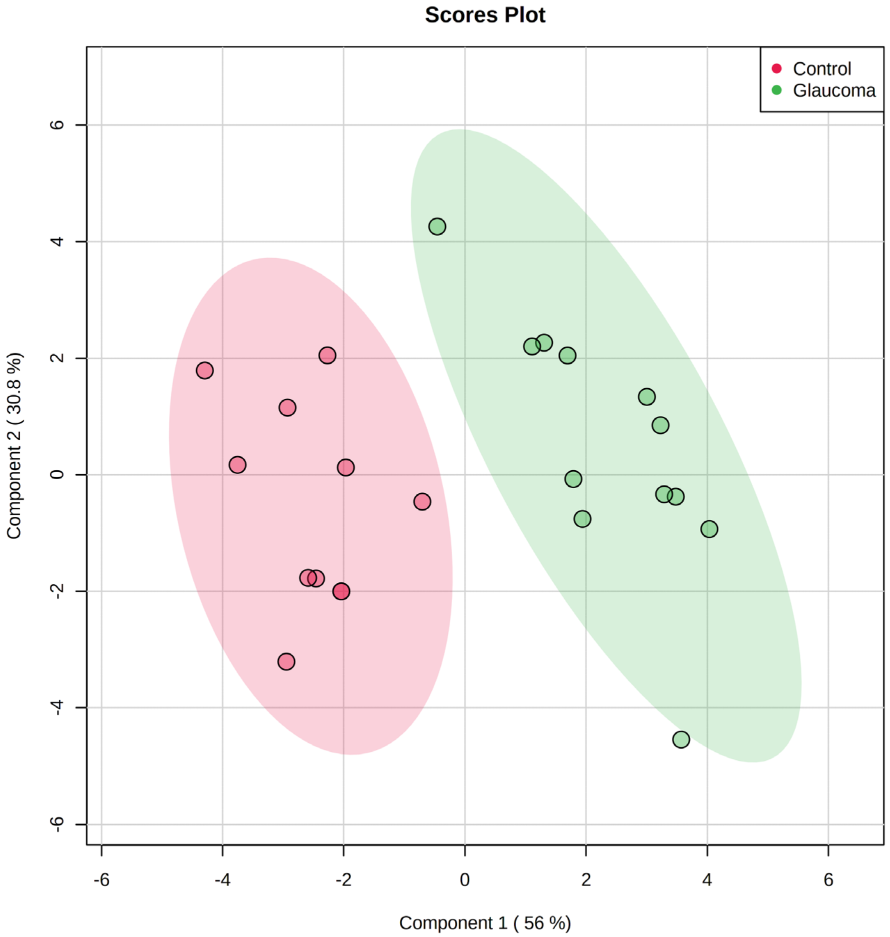

Supplemental Figure 4. Partial Least Squares Discriminant Analysis. Partial Least Squares Discriminant Analysis (PLS-DA) of total lysophospholipid (LPL) of optic nerve was created using Metaboanalyst 4.0. Mean peak intensity mass spectrometric data was quantile normalized and log2 transformed. The control and glaucoma groups are as indicated, and the two groups present as distinct clusters.

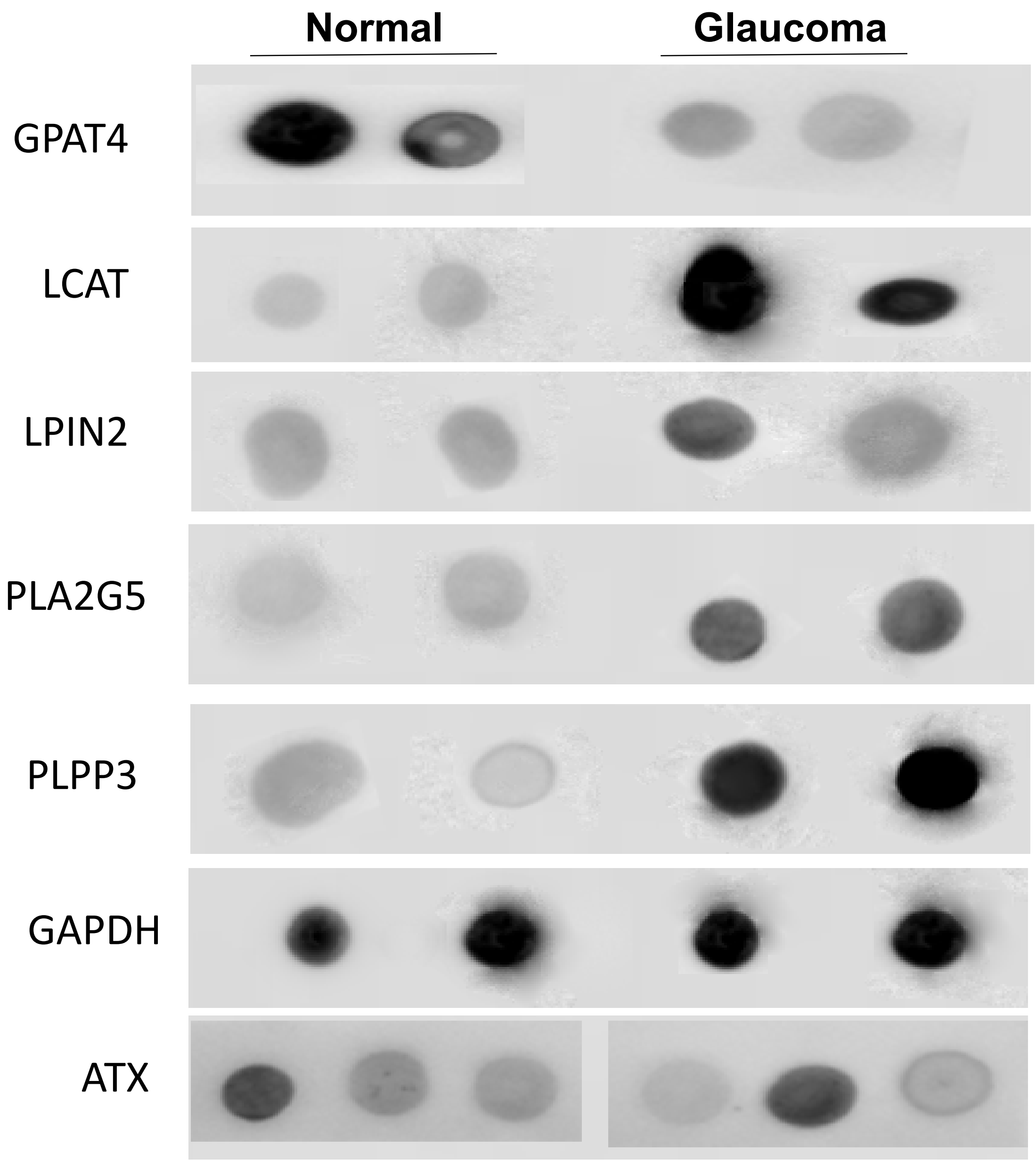

Supplemental Figure 5. Representative dot blot analysis. ON proteins (0.5μg) of normal or glaucoma donors as indicated were transferred on to PVDF membrane and probed with antibodies specific to the enzymatic proteins as indicated.
